# BRSK2 induced by nutrient deprivation promotes Akt activity in pancreatic cancer via downregulation of mTOR activity

**DOI:** 10.18632/oncotarget.17965

**Published:** 2017-05-18

**Authors:** Hexige Saiyin, Ning Na, Xu Han, Yuan Fang, Yanhua Wu, Wenhui Lou, Xianmei Yang

**Affiliations:** ^1^ State Key Laboratory of Genetic Engineering, School of Life Sciences, Fudan University, Shanghai 200433, People's Republic of China; ^2^ Department of Kidney Transplantation, The Third Affiliated Hospital of Sun Yat-sen University, Guangzhou 510630, People's Republic of China; ^3^ General Surgery Department, Zhongshan Hospital, Fudan University, Shanghai 20032, People's Republic of China

**Keywords:** BRSK2, PDAC, Akt, mTOR, AMPK

## Abstract

Neoplastic cells in pancreatic ductual adenocarcinoma (PDAC) survive in an energy-deprived milieu, and hyper-activation of Akt is thought to contribute to the neoplastic cell survival in PDAC. *Kras* activating mutations, common in PDAC, was believed to be the major driver of Akt activation. However, the inhibitor to Kras was not therapeutic for PDAC patients. This implied that PDAC cells might harbor an intrinsic merit that strengthens Akt activity. Here we showed that BRSK2, a serine/threonine-protein kinase of AMPK family, was induced by nutrient deprivation in PDAC cells and suppressed mTORC1 activity via phosphorylation of tuberous sclerosis complex 2 (TSC2). The suppression of mTORC1 activity in PDAC results in a dominant loss of feedback inhibition on Akt activity by mTORC1, consequently enhancing cell survival. This finding indicates that the intrinsic molecular merit that BRSK2 provides is a survival advantage to PDAC cells and strengthens the invasiveness of these neoplastic cells in energy-deprived environments.

## INTRODUCTION

PDAC, a highly lethal tumor, is rich in desmoplastic stroma and rare in microvasculature, which were considered to be the main barriers to nutrient uptake and drug delivery [1, 2]. Although PDAC accumulates the common mutations of epithelial tumors including *KRAS, p53, CDKN2* and *SMAD* [1, 3, 4], PDAC cells are more tolerant to nutrient deprivation and hypoxia than other epithelial-derived tumor cells, and are more resistant to chemotherapy as well [5]. The strong survival of PDAC cells depends on the activation of Akt, a critical survival kinase. Activating *KRAS* mutations (G12V or G12D), which occur in nearly a third of epithelial tumors and in nearly 90% of pancreatic cancers, are considered the major driver of consecutive Akt activation in PDAC tissues [4, 6]. However, the inhibitor to K-ras was not found to be therapeutic for PDAC [7–11]. Moreover, despite the energy-deprived milieu in PDAC, necrosis is rare in the tumor. These implied that PDAC cells might have some tissue-specific molecules that upregulate the activity of Akt to strengthen the tumor cell survival in nutrient-deprived milieu. But these tissue-specific characteristics are not well understood.

mTOR is an important player in sensing cellular energy and oxygen levels. mTOR integrates multiple signals from upstream pathways, including PI3K/AKT, and participates in regulation of transcription, protein synthesis, and cell growth and proliferation [12]. Normally, upon stimulation by various signals, PI3K/AKT can indirectly lead to an upregulation of mTORC1 activity via phosphorylation of the Tsc2 (tuberous sclerosis complex 2). Activated mTORC1 phosphorylates S6K (ribosomal protein S6 kinase) and 4EBP1 (eIF4E-binding protein), resulting in increased translation of proteins involved in cell cycle regulation [6, 13]. There exists a negative feedback to reduce PI3K/AKT signaling through S6K activation. When mTORC1 is active, S6K directly phosphorylates and inhibits IRS-1, which suppresses PI3K-AKT signaling [14]. This feedback inhibition was especially prominent in human non-malignant tumor hamartomas that harbor TSC2 mutation [15, 16]. TSC2 mutation caused a consecutive activation of mTORC1, which led to suppression of Akt activity and restricted malignant transformation of benign tumor cells [6, 13, 15]. AMPK, an upstream kinase of mTORC1, is another energy sensor, responding to changes in the cellular ATP/AMP ratio and participating in cellular energy homeostasis. AMPK directly phosphorylates Tsc2 on T1227 and S1345 [6], which stabilizes Tsc1-Tsc2 complex [17]. The inhibition of Rheb by Tsc complex is then enhanced, ultimately resulting in a decrease of mTORC1 activity [6]. In our previous work, we reported the AMPK member BRSK2, which is upregulated in PDAC and related to the malignant characteristics of PDAC [18]. But the mechanism by which BRSK2 is involved in PDAC has never been fully understood.

Here we reported that BRSK2, a kinase only expressed in the brain and normal pancreatic duct and islets, was found to be highly expressed in neoplastic PDAC cells. BRSK2 upregulation inhibits mTORC1 activity in a TSC2-mediated manner, which may lead to loss of mTORC1 brake on Akt activity and deteriorate the Akt hyperactivation in PDAC. Akt hyperactivation might provide a survival advantage to PDAC cells, and worsen their invasive behaviors in nutrient deprivation conditions.

## RESULTS

### BRSK2 is upregulated in neoplastic cells of PDAC and IPMN

**Figure 1:**
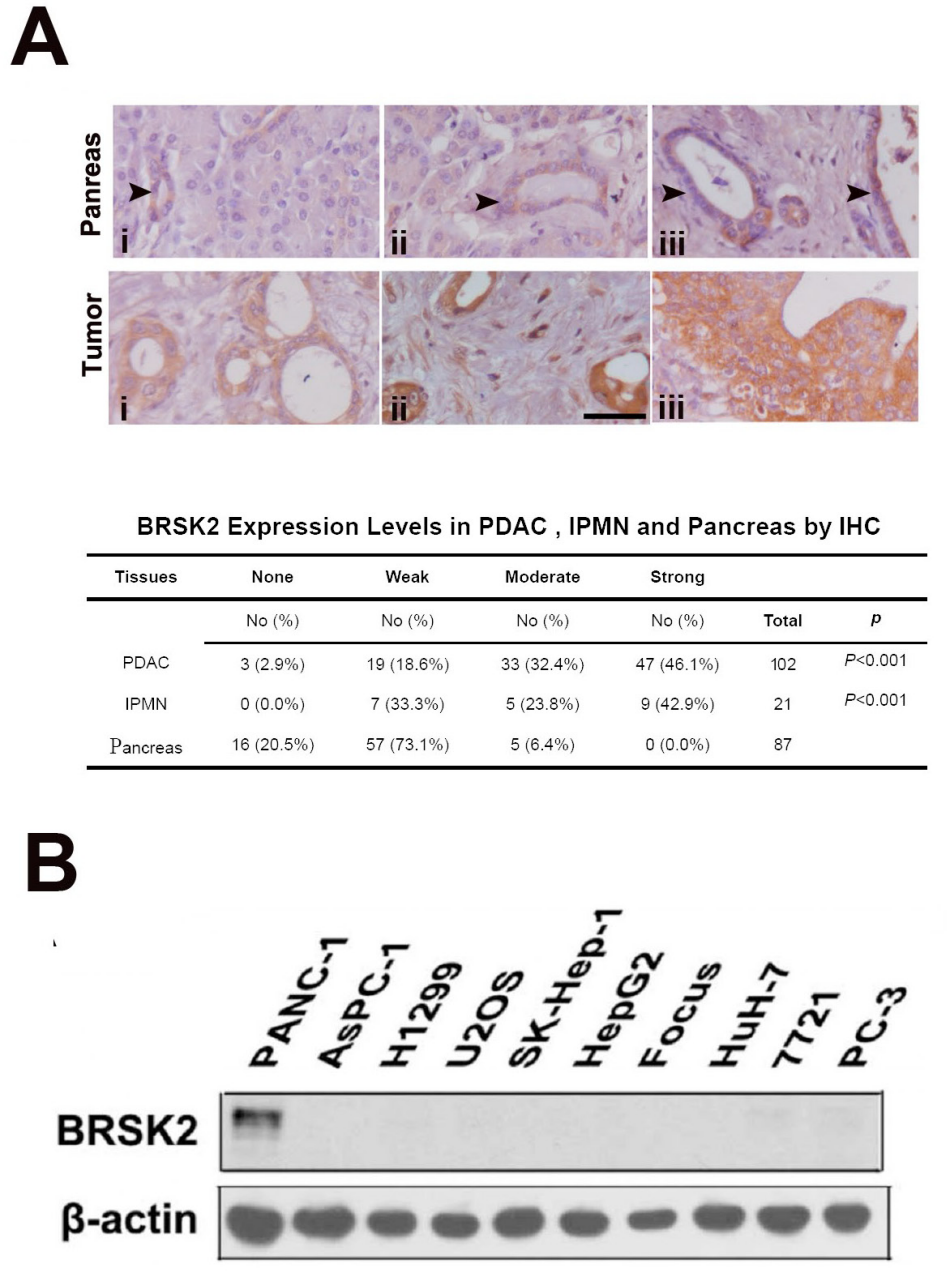
BRSK2 was upregulated in PDAC and IPMN tumors **(A)** BRSK2 expression patterns in pancreas and IPMN/PDAC tumor (tumor: i, IPMN; ii &iii, PDAC). Comparison of BRSK2 expression in PDAC and IPMN with non-tumor exocrine ducts. scale bar: 20 μm. **(B)** BRSK2 expression in an array of human cells including PANC-1, AsPC-1, H1299, U2OS, SK-Hep-1, HepG2, Focus, HuH-7, 7721, and PC-3.

We previously reported that BRSK2 is expressed in pancreatic islets and duct as well as in PDAC and regulates the secretion of insulin [18, 19]. To get a more comprehensive view on BRSK2 expression profiles in pancreatic cancers, we have expanded the PDAC cases from 79 to 102. Moreover, we also added the following cases to our current study: the intraductal papillary mucinous neoplasm (IPMN, the major precursor of PDAC; Supplementary Table 1), solid pseudopapillary neoplasm (SPN) and pancreatic neuroendocrine tumors (panNET), and hepatocellular carcinoma (HCC). Meanwhile, the follow-up time for all PDAC and IPMN patients had been extended to nearly 11 years. Consistent with our previous data [18], BRSK2 was only moderately expressed in the ductal system of exocrine part, such as intercalated duct, interlobular and intralobular duct, and pancreatic islet. BRSK2 was not detected in other cells in normal tissues, and was significantly upregulated in tumor cells of PDAC and IPMN (Figure 1A). However, BRSK2 was not detected in SPN and HCC, and was only weakly detected in panNET (data not shown). Our analyses showed that the BRSK2 expression levels in neoplastic cells are statistically correlated with clinical-pathological parameters such as vascular invasion and/or nerve invasion, but not with metastatic parameters including distance metastases and/or regional lymph node metastases (Table 1). Consistent with its tissue specific patterns, western blotting data showed that BRSK2 was expressed in PANC-1 cells, but was not detected in other tumor cell lines including liver cancer cell lines SK-Hep-1, HepG2, Focus, HuH-7, and SMMC-7721, lung cancer line H1299, osteosarcoma line U2OS, prostate cancer cell line PC-3, and another PDAC cell line AsPC-1 (Figure 1B). Survival and hazard factor analyses showed that BRSK2 was not directly correlated with survival in PDAC and IPMN patients, but it is a hazard factor for their survival. And this is especially significant in IPMN patients (PDAC+IPMN: HR, 1.390; IPMN: HR, 2.984; Table 2 and Supplementary Table 2).

**Table 1:** The correlations between BRSK2 and clinical pathological parameters in PDAC

		None	Weak	Moderate	Strong	p
Gender	Male	3	10	20	34	R=-0.012p>0.05
	Female	0	9	12	14	
Age	<60	2	5	15	18	R=-0.061p>0.05
	>60	1	14	17	30	
Tumor location	Head	2	18	25	30	R=0.260P=0.008
	Body-tail	1	1	6	13	
	Entire	0	0	1	5	
Tumor stage	Tis+T1+T2	1	5	8	7	R=0.143P=0.156
	T3+T4	2	14	23	41	
Regional lymph status	N.A	0	1	5	13	R=-0.166p>0.05
	No	3	8	8	19	
	Yes	0	10	19	16	
Distant metastases	No	3	18	28	46	R=-0.052p>0.05
	Yes	0	1	4	2	
Nerve invasion	No	2	9	9	7	R=0.340P=0.002
	Yes	1	10	23	41	
Vascular invasion	No	2	14	23	25	R=0.196P=0.050
	Yes	1	5	9	23	
Living status	lost	0	6	8	15	Log rank:p>0.05
	live	1	2	5	4	
	die	2	11	19	29	

**Table 2:** Hazard ratio of BRSK2 in IMPN+PDAC patients

Variables	Hazard ratio	95% CI
BRSK2 in tumor	1.390	0.794–2.435
pAkt substrate in tumor	1.499	0.579–3.878
MV fraction	0.732	0.437–1.225
MVD	0.740	0.433–1.236
Tumor stage	2.087	1.197–3.640
Regional lymph status	1.414	0.995–2.010
Tumor size	1.362	0.929–1.998
Nerve invasion	2.102	1.247–3.544
Vascular invasion	1.830	1.111–3.014

BRSK2 belongs to AMPK family, which has several important members including ARK5 and AMPK that are related to the aggressive behavior of tumors [20–22]. We further assessed the ARK5 and AMPK expression in PDACs and in normal pancreas by IHC, and found that ARK5 was exclusively expressed in vessels in both PDAC and normal pancreas tissues; AMPK was only expressed in stromal cells (Supplementary Figure 1). These data indicated that BRSK2 might be the exclusive member of AMPK which senses the nutrient supply in the milieu of PDAC and IPMN, and in ductal cells of normal pancreas.

### BRSK2 enhanced the survival of PDAC cells under nutrient deprivation

AMPK family members sense nutrient supply in milieu and are often upregulated upon energy deprivation [23]. To test whether BRSK2 expression was related to the nutrient supply of PDAC, we have compared the expression of BRSK2 in tumor tissues with microvasculature parameters which reflect the energy supply in tumors [24, 25]. Consistent with the nutrient dependent upregulation of AMPK family protein, BRSK2 expression level is inversely related to microvessel density (MVD) and microvessel coverage fraction in pancreatic cancer (Supplementary Figure 2A and 2B; r= -0.349, p<0.001; r=-0.255, p<0.008). *In vitro*, gradient 2-Deoxy-D-glucose (2-DG) treatment which mimics the effect of intracellular glucose deprivation induced BRSK2 upregulation in PANC-1 cells (Supplementary Figure 2C). Those data demonstrated that BRSK2 is a typical AMPK family member that senses the nutrient supply in PDAC tumor milieu.

**Figure 2:**
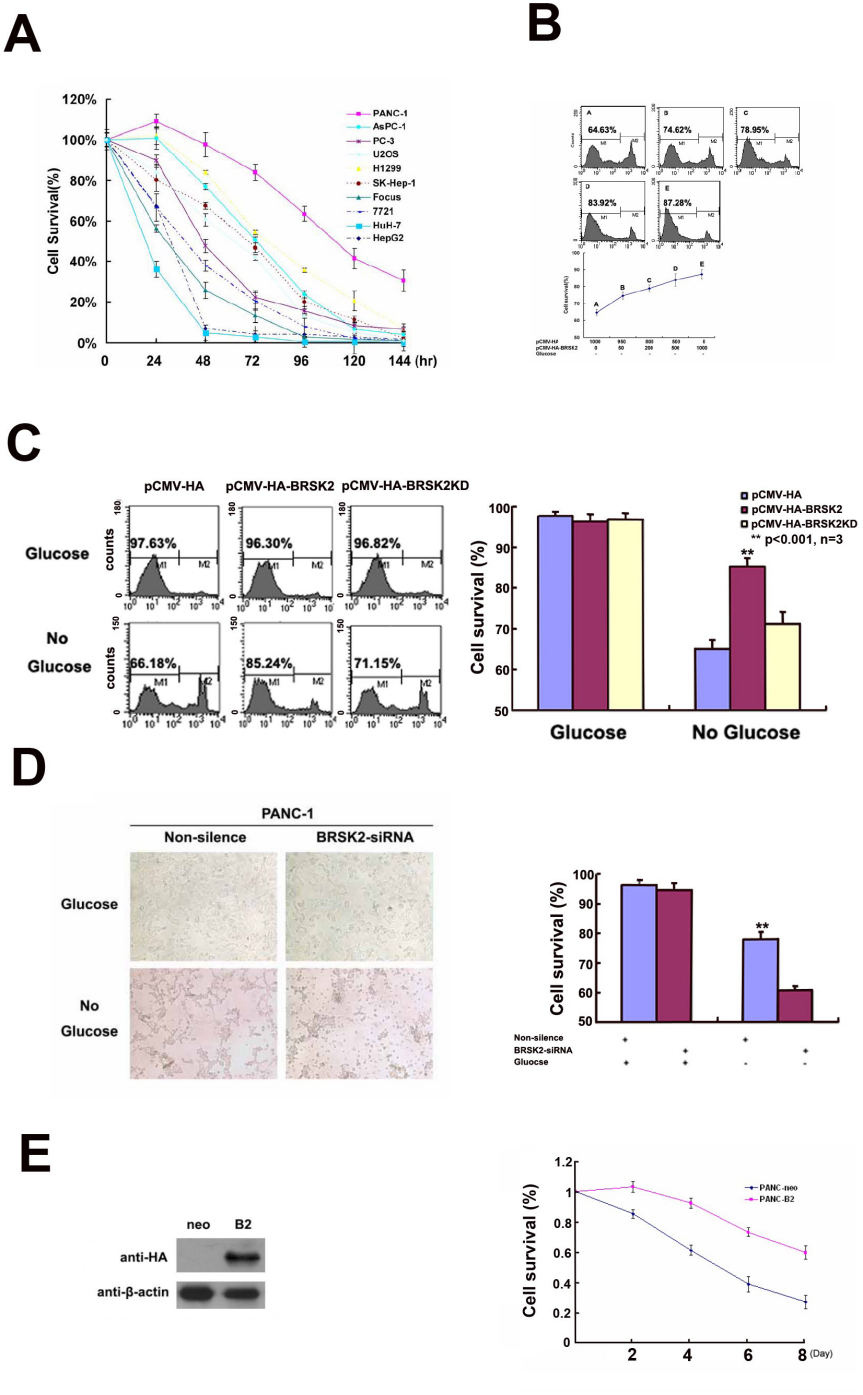
BRSK2 expression enhances cancer cells survival *in vitro* **(A)** Cell survival ability of different cells upon nutrient deprivation. Different cells were subjected to no-glucose culturing media, and survival assay (MTS/PMS) was performed at appropriate time points (24-144 Hours). **(B)** Overexpression of BRSK2 increased cell survival under conditions of nutrient deprivation. PANC-1 cells were transiently transfected with series amount of HA-BRSK2 constructs. After 72 hours of no-glucose treatment, cells were stained with PI and subjected to FACS for survival assay. And the cell survival rate increased along with the amount of exogenously expressed BRSK2. **(C)** Effects of BRSK2 on cell survival during nutrient deprivation. Cells were transiently transfected with constructs exogenously expressing BRSK2 (or kinase-dead mutant BRSK2KD). Then the cells were subjected to no-glucose culturing media for 72 hours before performing the cell survival assay (FACS assay). **(D)** Knock-down of endogenous BRSK2 expression decreased cell survival upon nutrient deprivation. PANC-1 cells were transiently transfected with siRNA fragment specifically targeting BRSK2, and the cells were recorded with microscope 48 hours after no-glucose treatment. **(E)** Stable over-expression of BRSK2 increased cell survival under conditions of nutrient deprivation. Mono-clone PANC-1 cell line stably expressing HA BRSK2 was constructed (neo as the control cell line). The stable cell lines were seeded in 96-well plates, and were subjected to no-glucose culturing media. The survival assay (MTS/PMS) was performed at appropriate time points (n=6).

**Figure 3:**
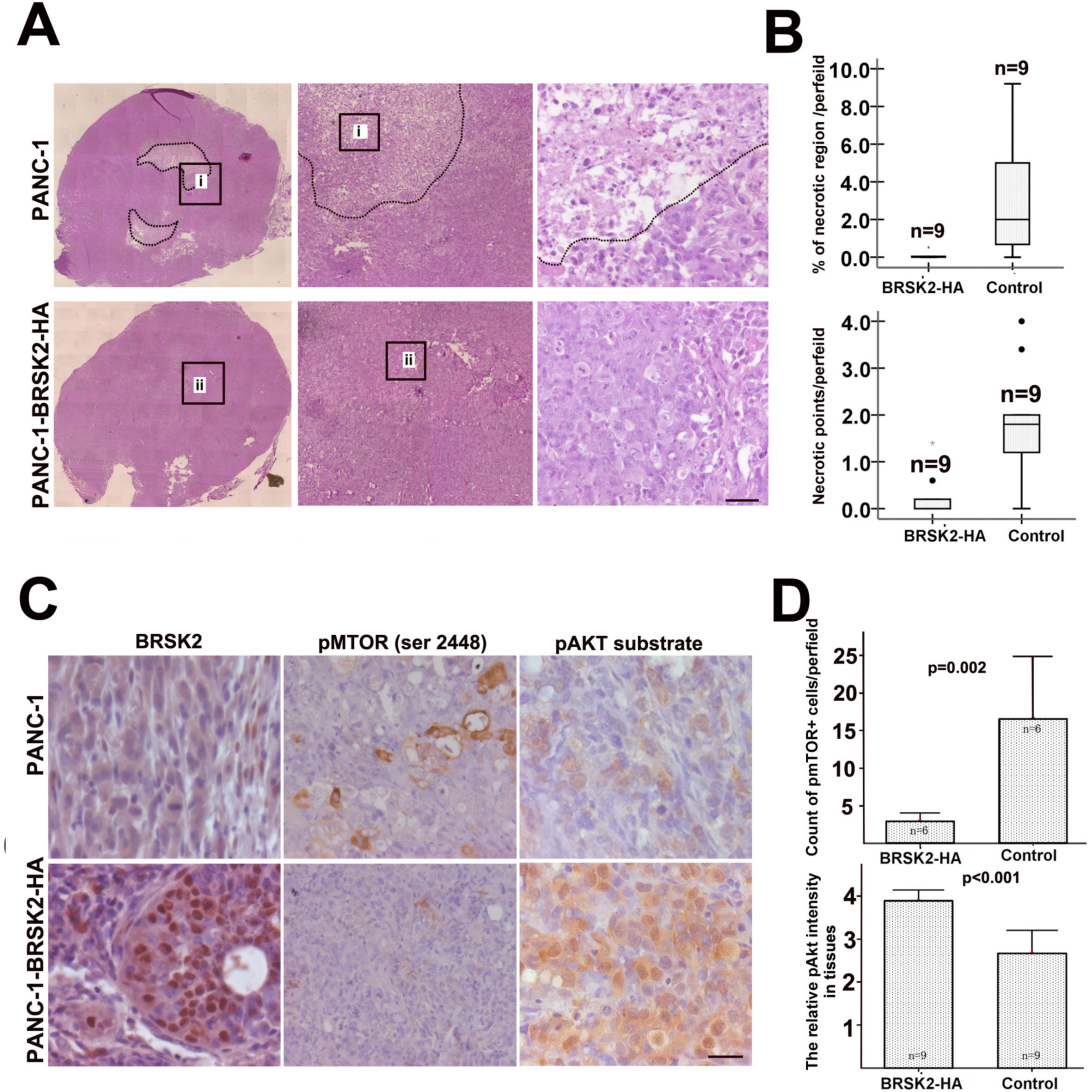
BRSK2 expression strengthened cell survival *in vivo* and activated Akt **(A, B)** Histology showed the necrosis in the core of xenograft tumors from PANC-1 highly expressing BRSK2 and control PANC-1. Scale bar: 40 μm. **(C, D)** IHC of pmTOR (Ser 2448) and pAkt substrate in xenograft tumors from BRSK2-overexpressing PANC-1 and control PANC-1. Scale bar: 20 μm.

Furthermore, we exposed different cells to glucose starvation. BRSK2-expressing cells are more tolerant to glucose starvation than cells that do not express BRSK2 (Figure 2A). To further test the role of BRSK2 in cell survival, we constructed the pCMV-HA-BRSK2 vector and transfected it into PANC-1 cells, and found that increasing pCMV-HA-BRSK2 dosage enhanced the survival of PANC-1 under glucose starvation (Figure 2B). When we expressed the kinase-dead mutant BRSK2-KD in PANC-1 with gradient to compete with endogenous BRSK2, a decrease of survival cells with gradient was observed (Figure 2C). Knockdown of BRSK2 by RNAi significantly decreased the tolerance of PANC-1 to glucose starvation (Figure 2D). In addition, we have constructed stable BRSK2-expressing PANC-1 cell line, and exposed the cells to low or no glucose media. The stable BRSK2 high-expression cell line was more resistant to glucose starvation than were normal cells (Figure 2E). To test the *in vivo* effect of BRSK2 expression in PDAC, we also transplanted the stable BRSK2-expressing cells into nude mice, and found that high expression of BRSK2 had no effect on the growth rate of xenograft tumor. However, we observed more necrosis in control tumor by the H&E staining. The measurement data of necrotic region showed not only that the necrotic size in BRSK2 stable expressing xenografts is significantly smaller than that in the control, but also that the necrotic points are fewer than that in control (Figure 3A and 3B). These findings further demonstrate that BRSK2 has an ability to enhance PDAC cell survival under nutrient deprivation conditions *in vitro* and *in vivo*.

Hyper-activation of mTORC1 was reported to inhibit Akt activity by feedback loop in benign tumor-hamartoma harboring TSC2 mutation [15]. This inhibition of Akt by mTORC1 is often dominant and prevents benign tumor from malignant transformation [6]. Under nutrient-deprived condition, AMPK family members often repress the mTOR activity [17]. Along with our previous BRSK2 data, this implied that BRSK2 upregulation in pancreatic cancer might enhance the activity of Akt via mTORC1 inhibition. To test this speculation, we first tested mTORC1 activity with pmTOR (Ser 2448) antibody and Akt activity with its substrate phosphorylation antibody in our xenograft tissues by IHC. The results showed that pmTOR levels in BRSK2-HA xenografts are lower than that in the control, while phospho-Akt substrate levels are higher than that in control xenografts (Figure 3C and 3D). These data implied that nutrient deprivation-induced BRSK2 expression might enhance the survival of human pancreatic cancer cells by enhancing Akt activity.

### BRSK2 expression in PDAC patient tissues might dominantly induce loss of feedback inhibition on Akt activity by mTORC1

**Figure 4:**
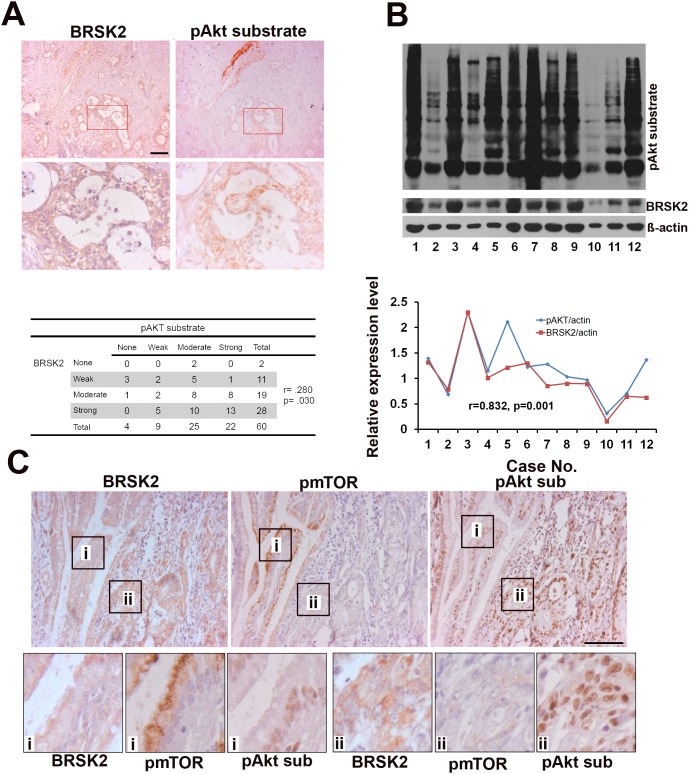
The relationship of BRSK2, pAkt substrate and pmTORS (2448) in cancer cells of human PDAC tissues **(A)** BRSK2 and pAkt substrate expression pattern in PDAC and the correlation of BRSK2 and pAkt substrate expression by IHC. Scale bar, 100μm. **(B)** The detection of BRSK2 and pAkt substrate expression levels in PDAC tissues with western blotting. The expression levels of BRSK2 and Akt activity were correlated in PDAC tissues (n=12). **(C)** BRSK2, pAkt substrate and pmTOR (Ser2448) expression pattern in consecutive sections of PDAC tissues. Scale bar, 200μm.

Similar to other solid tumors, human PDAC is heterogenic and differs from xenograft tumor [26]. Here, to see whether BRSK2 upregulation relates to Akt in human PDAC, we tested the relationship of BRSK2 expression with Akt activation in the human PDAC tissues. We have detected Akt activity with pAkt substrate antibody that reflects total Akt activity by IHC and western blotting. Both IHC and western blotting data showed that BRSK2 levels in the tumor tissues are positively correlated with pAkt substrate intensity (Figure 4A and 4B; r=0.280, p=0.030; r=0.832, p=0.001). Therefore, there exists the possibility that BRSK2 dominantly activates Akt in human PDAC.

Despite mTORC1 is considered to be a target for solid tumor therapy including PDAC, mTORC1 inhibition often leads to hyperactivation of Akt [27, 28]. To get a comprehensive view on mTORC1 activation in PDAC, we have tested mTORC1 activity using phospho-mTOR (Ser2448) antibody in PDAC tissues. Contrary to liver and PANC-1 cells, we didn't detect pmTOR in PDAC tissues by western blotting (Supplementary Figure 3A), and observed only sporadic phospho-mTOR positive cells in PDAC tissues by IHC (Supplementary Figure 3C). We have further done a more precise comparison of the expression patterns of these proteins in 5μm-thick consecutive slides, which often show one cell in two slides. Surprisingly, a positive correlation of BRSK2 with pAkt substrate signal and a negative correlation of pAkt substrate signal with pmTOR signal were observed in a dozen consecutive PDAC sections (Figure 4C). Contrary to pancreatic islets (Supplementary Figure 3B), the negative correlation of pAkt substrate signal and pmTOR signal were widely existed through both PDAC tissues and harmatoma (Supplementary Figure 3B and 3C). These findings implicate that losing the feedback inhibition on Akt by mTORC1 might be another dominant way to activate Akt in human PDAC tissues.

### BRSK2 enhanced Akt activity via repressing mTORC1 in PANC-1 cells

**Figure 5:**
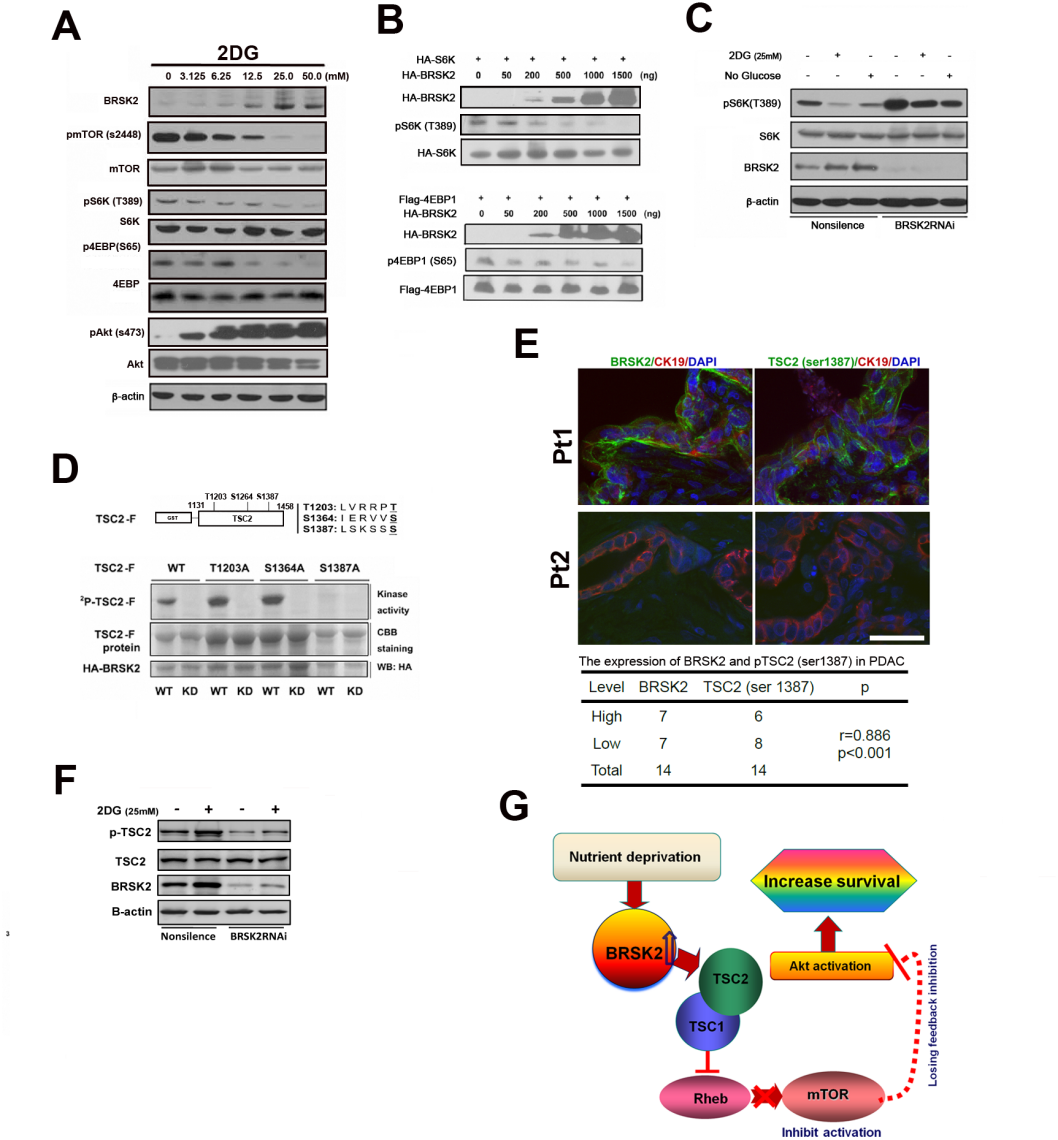
BRSK2 in PDAC cells strengthens Akt activity via TSC2-dependent mTORC1 repression **(A)** Effect of 2-DG treatment on BRSK2 and other components of AKT/mTOR signaling pathway. PANC-1 cells were treated with series concentrations of 2-DG for three hours before analysis. **(B)** Overexpression of BRSK2 inhibited S6K and 4EBP-1 phosphorylation. PANC-1 cells were transiently co-transfected with pCMV-HA-BRSK2 and pCMV-HA-S6K (or pFLAG-CMV4-4EBP1) constructs. **(C)** Inhibition of S6K phosphorylation upon energy deprivation was alleviated when BRSK2 was knocked-down. PANC-1 cells were first transiently transfected with BRSK2 specific siRNA fragment or control fragment. 3 days after transfection, cells were treated with regular media plus 2-DG or no glucose media for three hours. **(D)** BRSK2 phosphorylated TSC2 on Ser1387 *in vitro*. **(E)** BRSK2 expression levels are positively correlated with the levels of TSC2 (Ser1387) in human PDAC. We have co-immunostained the consecutive frozen sections of human PDAC samples (n=14) with pTSC2 (Ser1387) or BRSK2 and Cytokeratin. Scale bar: 50 μm. **(F)** Activation of TSC2 phosphorylation upon energy deprivation was impeded when BRSK2 was knocked-down. PANC-1 cells were first transiently transfected with BRSK2 specific siRNA fragment or control fragment. 3 days after transfection, cells were treated with regular media plus 2-DG for three hours. **(G)** Scheme of BRSK2's role on Akt activation in PDAC. BRSK2 was significantly upregulated in the nutrient deprivation micro-environment, which inhibited mTOR via phosphorylating TSC2. This repression of mTOR may cause a loss of feedback inhibition on Akt activation. And hyperactivation of Akt possibly would attribute to increased survival in PDAC.

Based on our observation of the dominant induction of Akt activity by BRSK2 via repression of mTOR in human PDAC samples, we tested whether this activation pathway worked in PDAC. To test this pathway, we rendered PANC-1 cells to 2-DG treatment. Upon gradient 2-DG treatment, induction of BRSK2 was synchronously observed with repressed levels of pmTOR (Ser2448), pS6K (Thr389) and p4EBP (Ser65), as well with increased level of pAkt (Ser473) (Figure 5A). This is consistent with our previous result in human PDAC tissues. We also expressed both BRSK2 and S6K (or 4EBP1) exogenously in PANC-1 cells, and the phosphorylation of S6K (or 4EBP1) was inhibited with the increasing level of BRSK2 (Figure 5B). Meanwhile, inhibition of S6K activity by 2-DG treatment was hindered upon BRSK2 knockdown by RNAi (Figure 5C). All these suggested that the decrease of S6K and 4EBP1 activity might be attributed to the increase of BRSK2 level, and were consistent with our previous postulation that the upregulation of BRSK2 eventually results in loss of feedback inhibition on Akt via mTORC1 pathway.

### BRSK2 modifies mTORC1 activity via phosphorylation of TSC2

TSC2 is a GTPase-activating protein (GAP), which is rarely mutated in PDAC [29], and its mutation often causes a benign tumor. TSC2 inactivates the Ras-like GTPase Rheb, indirectly suppressing mTOR activity. While TSC2 can be inactivated via phosphorylation by Akt, phosphorylation of TSC2 with AMPK enhances its GAP activity, leading to mTOR inhibition [6]. Therefore, it is possible that the observed modulation of mTORC1 activity upon BRSK2 upregulation is via TSC2. Indeed, *in situ* immunofluorescence staining with tumor sample displayed colocalization of these two proteins in the tumor cells (Supplementary Figure 4B). Furthermore, *in vitro* immunoprecipitation showed that TSC2 and BRSK2 could interact with each other (Supplementary Figure 4A). These results showed that TSC2 has the potential to interact with BRSK2 in tumor cells of PDAC tissues.

BRSK2 preferentially phosphorylates the consensus sequence LxRxxS/T. Here x stands for any amino acid, and L could be substituted by M, I, F or V. Moreover, R could be replaced with K or H with the order of R>K>H [30]. TSC2 protein contains 9 such conserved motifs. While multiple phosphorylation sites have been identified on TSC2, the phosphorylation at Ser1387 have been confirmed to participate in promoting its GAP activity towards Rheb [31]. To decide if BRSK2 also phosphorylate this site, we cloned the sequence of TSC2 containing Ser1387 (from amino acids 1131 to 1458, harboring three consensus sequences) into pGEX-6p-1 vector. The prokaryotically expressed fusion protein was named TSC2-F. The HA-tagged wild type kinase BRSK2 (WT; with the mutant BRSK2-KD being the negative control) was exogenously expressed in mammalian cells. We also mutated all three potential phosphorylation sites one by one in the TSC2-F fragment clone (Figure 5D). Our *in vitro* kinase assay revealed that phosphorylation of the protein fragment was inhibited only when Ser1387 was mutated, indicating that BRSK2 could phosphorylate TSC2 at Ser1387. We further investigated the role of BRSK2 in regulation of TSC2 Ser1387 phosphorylation in cells. With the treatment of 2-DG, BRSK2 expression level increased, so as the phosphorylation of TSC2 at Ser1387. And this increasing of TSC2 phosphorylation by 2-DG treatment was impeded upon BRSK2 knockdown by RNAi (Figure 5F). Moreover, our Immunohistochemical staining assay with tumor samples further revealed a close correlation between BRSK2 protein level and TSC2 Ser1387 phosphorylation level (Figure 5E). As TSC2 mutation in PDAC is rare [3, 29], it is likely that BRSK2 downregulates mTORC1 through phosphorylation of TSC2.

## DISCUSSION

The milieu of pancreatic tumors is characterized by heavy fibrosis, severe hypoxia, and low nutrient supply [32]. To survive in this milieu, PDAC cells shift metabolism from aerobic to anabolic, enhanced glycolysis [33, 34], and hyperactivated Akt. These survival advantages of PDAC are considered to be driven by KRAS activating mutation, but Kras inhibitor was not found to be therapeutic in PDAC patients. In this work, we showed that the low-nutrient milieu of PDAC might provide a crucial signaling to Akt activation through upregulation of BRSK2, an AMPK catalytic subunit family member exclusively expressed in pancreas and PDAC cells, enhancing survival of PDAC cells. BRSK2-induced loss of feedback inhibition on Akt activity in nutrient-deprived condition might deteriorate Kras-induced Akt activation, and provide more survival and behavioral advantages to PDAC cells than other cells which do not possess such a tissue-specific molecular merit (Figure 5G).

mTOR is an important component of PI3K/AKT/mTOR pathway [23]. It is well documented that hyperactivation of Akt indirectly increases mTOR activity via inhibitory phosphorylation of TSC2. Indeed, mTORC1 had been reported to be over-activated in many cancers as a result of increased activity of PI3K or Akt [35]. However, it has been noted that patients suffering from tuberous sclerosis complex (TSC) do not develop more aggressive tumors like those linked to mutations in *PTEN* [6], which functions upstream of PI3K/AKT. The underlying rationale may be the existence of a potent negative feedback loop, where activation of mTORC1 strongly represses upstream PI3K/AKT signaling [36]. Genetic evidence in mice supports this hypothesis, as inactivation of *PTEN* in tsc2-deficient lesions elevates AKT signaling sufficiently to overcome the feedback loop and results in more severe tumors [37]. This indicates that mTORC1 may, to some extent, suppress PI3K-AKT signaling and function as a brake for Akt. mTORC1 also senses cellular nutrient/energy and oxygen levels [38], and functions downstream of AMPK, a sensor of cellular energy balance [23]. Upon energy depletion, AMPK is activated, and results in mTORC1 inhibition. Therefore, the inhibition of mTORC1 by energy depletion might release the brake for Akt, providing an advantage in cell survival. Human cells in different organs live in different milieu from oxygen supply to nutrient availability. Thus, different cells adopt different strategies to survive in energy-deprived conditions or other harsh environments. Based on exclusive expression of BRSK2 in PDAC, we speculated that upregulation of BRSK2 in PDAC might lead to the loss of feedback inhibition on Akt by mTORC1. Our *in vitro* and *in vivo* data showed that up-regulation of BRSK2 not only strengthened the cellular survival of PANC-1, but also decreased mTORC1 activity and enhanced Akt activity. This Akt activation is achieved by a traditional AMPK pathway similar to ARK5, which decreased the necrosis in tumor xenografts [21]. In addition, human PDAC sample analyses showed that BRSK2-mediated repression of mTORC1 was significant, and activation of Akt was prominent in human PDAC tissues. The relation of BRSK2 expression level with invasiveness in PDAC patients further showed that BRSK2 has a potential to deteriorate the progress of PDAC. Furthermore, our long-term follow-up data in IPMN patients showed that BRSK2 also has a potential to accelerate the progression of IPMN, the early precursor of PDAC. This finding implied that besides tumor driving mutations, tissue-specific merit might provide more advantages for PDAC cell survival in nutrient deprived condition.

Taken together, our work here revealed a tissue-specific routine by which pancreatic tumor cells hyper-activate Akt under nutrient-deprived conditions. This finding provides an explanation for why tumors formed in different organs differ in cell survival and drug sensitivity, even though they are driven by common mutations. Besides tumor-specific mutations, tissue-specific characteristics also need to be considered or included in therapeutic design.

## MATERIALS AND METHODS

### Constructs, siRNA fragments, antibodies, and tumor samples

The plasmids used for cloning in this study are pCMV-HA (Clontech), pFLAG-CMV4 (Sigma), and pGEX-6P-1 (GE Healthcare). The stable cell line used for mouse xenograft model was established with construct pCMV-HA-BRSK2 in PANC-1.

The sequence of the siRNA fragment specifically knock-downing BRSK2 expression is as follow: 5’-GCTAGAGCACATTCAGAAA-3’. Effect of siRNA was verified with RT-PCR and western blotting.

For BRSK2 antibody, BRSK2 gene was cloned into pET-28a vector. The His-tagged BRSK2 protein was induced and purified to immunize rabbit for polycolonal antibody production. The specificity and efficiency of the antibody were verified.

This study enrolled 127 patients with pancreatic tumors, including 102 cases of PDAC, 21 cases of intraductal papillary mucinous neoplasm (IPMN), 2 cases of neuroendocrine tumor (NET), and 2 cases of solid pseudopapillary neoplasm (SPN). All had undergone tumor resection at the General Surgery Unit of Zhongshan Hospital affiliated to Fudan University, between January 2005 and September 2007 (Supplementary Table 1). Samples from 57 patients contained pancreatic tissues that were morphologically normal. The staging of the tumors was performed according to the Standards of the American Joint Committee on Cancer 2.0. And all the procedures were approved by the Medical Ethics Committee of Zhongshan Hospital.

### Cell lines, cell culturing and energy deprivation treatments

The cell lines used in this study are PANC-1, AsPC-1, H1299, U2OS, SK-Hep-1, HepG2, Focus, HuH-7, SMMC-7721, and PC-3. Focus and SMMC-7721 were kindly provided and licensed by Cell Bank of Institute of Biochemistry and Cell Biology, Shanghai Institutes for Biological Sciences. And all the rest were from ATCC. The cell lines were grown in Dulbecco's Modified Eagle's Media (DMEM) or RPMI-1640 media accordingly, supplemented with heat-inactivated 10% fetal bovine serum (Gibco BRL). All the cells were maintained at 37°C in the atmosphere of 5% CO_2_.

2-DG used was purchased from MedChem Express (MCE), and dissolved in distilled water at 1M as stocking solution. Before 2-DG treatment, cells were washed twice with PBS, and culturing media containing appropriate concentration of 2-DG were added then. The 2-DG treatment usually lasts for three hours before rendering the cells for following analysis. For glucose starvation experiments, culturing media containing no glucose was added to cells after PBS washing, and the cells were collected at proper time points for cell survival test.

### Cell survival assay

Two survival assays were employed here. After treatment, cells were collected and washed before staining with PI (final concentration = 50 μg/ml). After staining with PI for 3 min at dark, cells were subjected to FACS analysis. The live cells, not being stained with PI, attribute to M1 peak (background signals). The M2 peak was attributed to the real fluorescent signal of PI staining (of dead cells).

The second survival assay utilized MTS/PMS method. Cells were seeded in 96-well plates and were cultured with no-glucose media. For control, regular media was used. At certain time points, MTS/PMS solution was added to the cells according to the manufacture's protocol. Cell survival was presented as OD490 of treatment well / OD490 of control well. The number for the duplicated experiments was 6.

### Immunohistochemical staining and image analysis

The immuohistochemical staining was done following the protocol described in our previous work [25].

For double fluorescent immunostaining, slides were blocked in 10% goat/donkey serum after being deparaffined in xylene and rehydrated in alcohol. Antigen retrieval was achieved by microwave heating in a citrate acid buffer. Slices were incubated with a primary antibody at 4°C overnight, washed 3 times in PBS, and incubated at room temperature with a fluorescent-labeled secondary antibody (goat anti-mouse Alex 488 or 555 at 1:100 dilution) for 90 minutes.

Images were obtained with a Leica DMRI microscope (Leica, Germany) installed on a Lexica DC 500 camera (Leica Corporation, Germany). The digital video camera was connected to a personal computer loaded with Leica QWin Software (Leica, Germany). Image analyses were done as described in our previous work [25].

### Measurement of necrosis

The xenograft tumors were divided to two parts by the middle line. One part is fixed by 4% PFA for histological analyses, the other for protein and RNA extraction. We have sliced the tissues from the middle of tumor. 5 slices were used for measuring the necrotic region, and the distance between each slice is 80 μm. The slices were examined by two pathologists in double-blinded manner.

### Statistical analysis

The difference between categorical variables was assessed using the one-way Anova test and Spearsman correlation. Survival curves were estimated by the Kaplan-Meier method; the group was divided by median value, and differences were assessed by the log-rank test, hazard rario were analyzed by Cox's proportional hazards regression model. The clinical pathological variables included in this analysis were: gender; age; tumor loci (pancreas head, body-tail, entire pancreas); tumor stages (Tis, T1, T2, T3, T4); lymphatic invasion (yes vs no); metastasis (yes vs no); vascular invasion (yes vs no); nervous invasion (yes vs no).

## SUPPLEMENTARY MATERIALS FIGURES AND TABLES


