# Supplementary material for: BRSK2 induced by nutrient deprivation promotes Akt activity in pancreatic cancer via downregulation of mTOR activity
Source: Oncotarget. 2017 May 18;8(27):44669–81. doi: 10.18632/oncotarget.17965 (PMC5546509; doi:10.18632/oncotarget.17965)
Supplement: Supplementary file 1 [file oncotarget-08-44669-s001.pdf]

BRSK2 induced by nutrient deprivation promotes Akt activity in pancreatic cancer via downregulation of mTOR activity

SUPPLEMENTARY MATERIALS

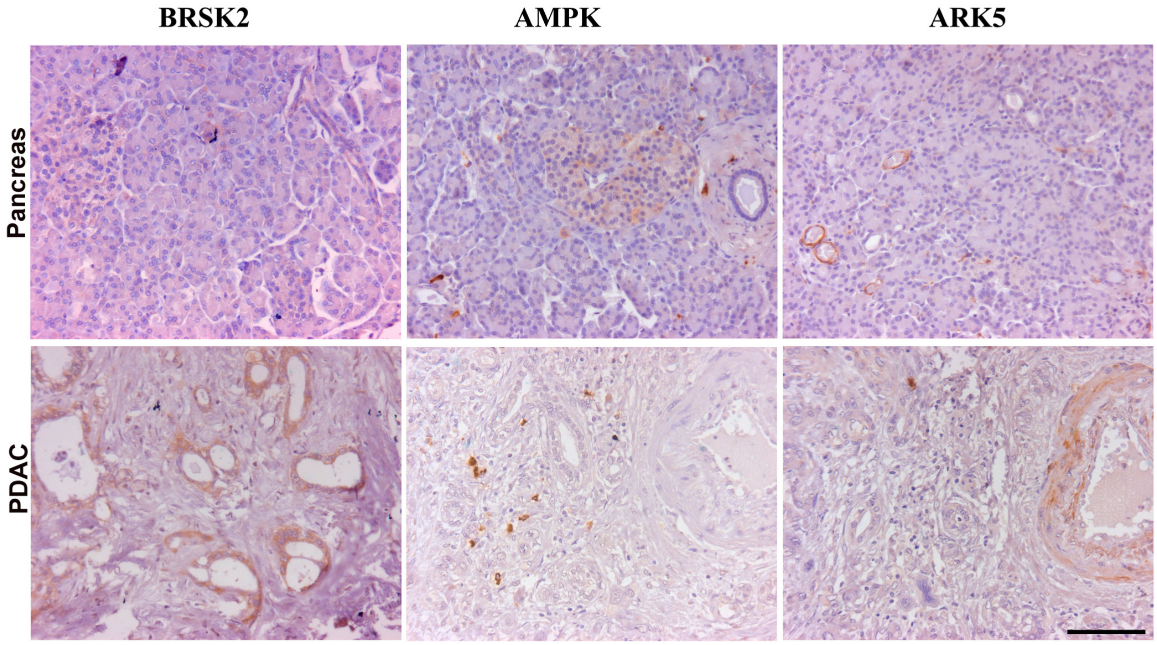

Supplementary Figure 1: The expression patterns of BRSK2, ARK5 and AMPK in normal pancreas and in PDACs. Scale bar, 200µm.

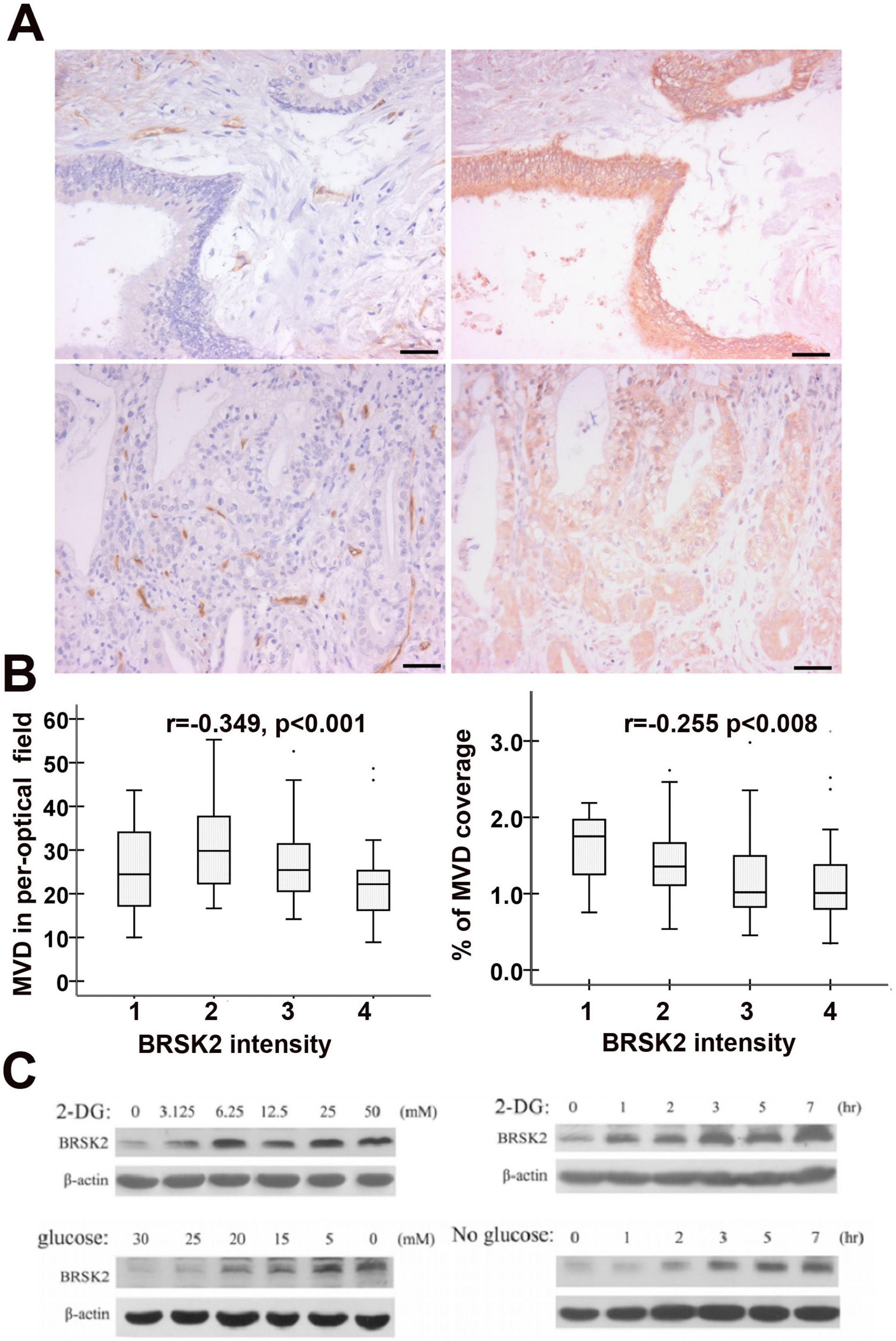

Supplementary Figure 2: BRSK2 is a typical AMPK family member that senses the nutrient supply in PDAC tumor milieu. (A and B) Representative images of CD34 and BRSK2 staining in two cases of PDAC. BRSK2 expression level is inversely related to microvessel density (MVD) and microvessel coverage fraction in pancreatic cancer (r= -0.349, p<0.001; r=-0.255, p<0.008). Scale bar, 50µm. **(C)** *In vitro* treatments of glucose deprivation induced BRSK2 upregulation in PANC-1 cells.

A

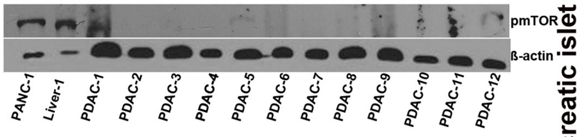

Table S3. BRSK2 and pmTOR (s2448) expression in PDAC patients

		BRSK2				
		0	1	2	3	Total
pmTOR (2448 ser)	0	0	3	2	4	9
	1	0	2	5	6	13
	2	1	4	7	8	20
	3	0	3	3	6	12
	4	0	2	2	5	9
	Total	1	14	19	29	63

pmTOR (2448 ser) IHC scoring: 0=no positive cells, 1=1~5 positive cells, 2=6~15 positive cells, 3=16~40 positive cells, 4= 40<positive cells, spearman correlation: $p>0.05$

B

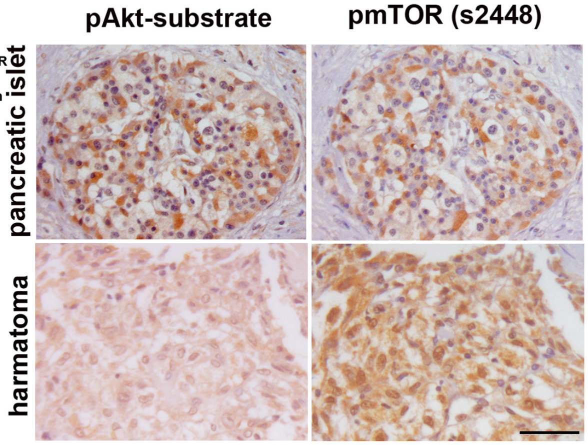

C

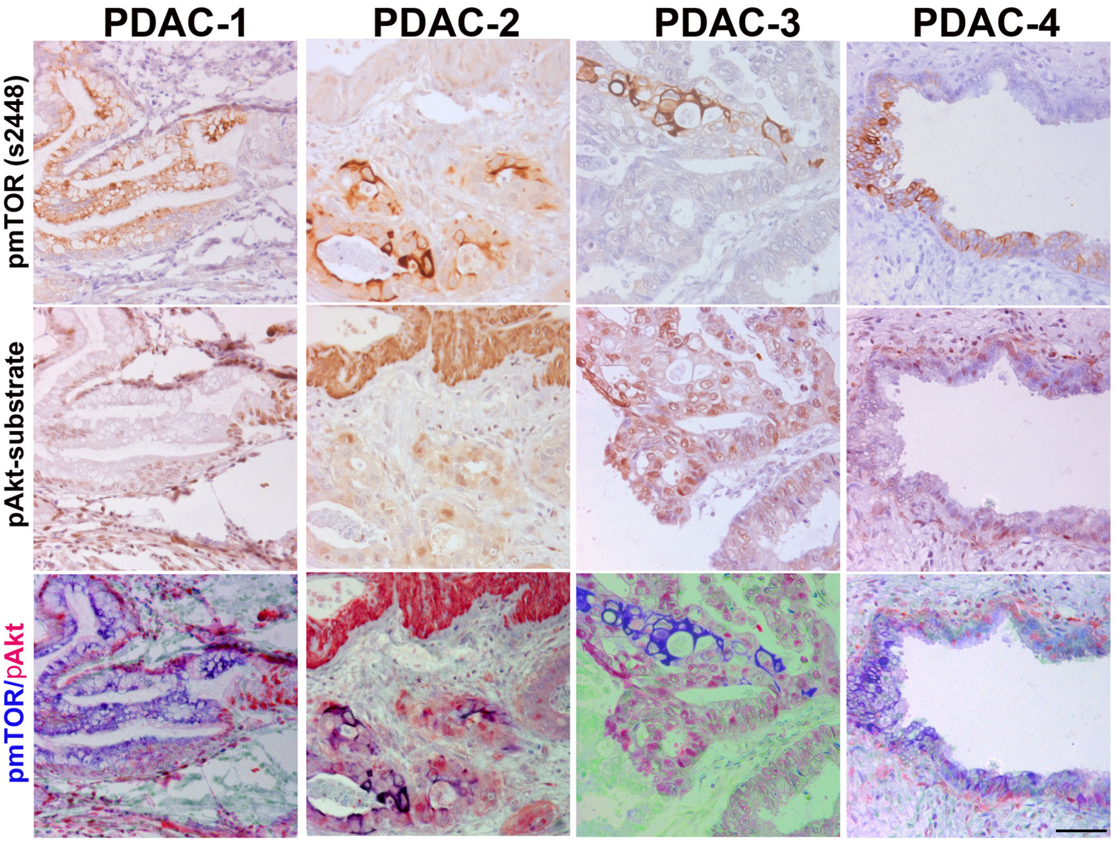

Supplementary Figure 3: Levels of pAkt substrate signal and pmTOR signal were negatively correlated in PDAC tissues. **(A)** The detection of pmTOR (Ser2448) expression in PDAC patients tissues by western blotting and IHC. Contrary to liver and PANC-1 cells, no expression of pmTOR was detected in PDAC tissues by western blotting, **(B and C)** pmTOR (Ser2448) and pAkt substrate expression patterns in consecutive sections of pancreatic islets, harmatoma tissues **(B)** and four cases of PDAC **(C)**. Contrary to pancreatic islets, the negative correlation of pAkt substrate signal and pmTOR signal were widely existed through both PDAC tissues and harmatoma. scale bar, 100µm.

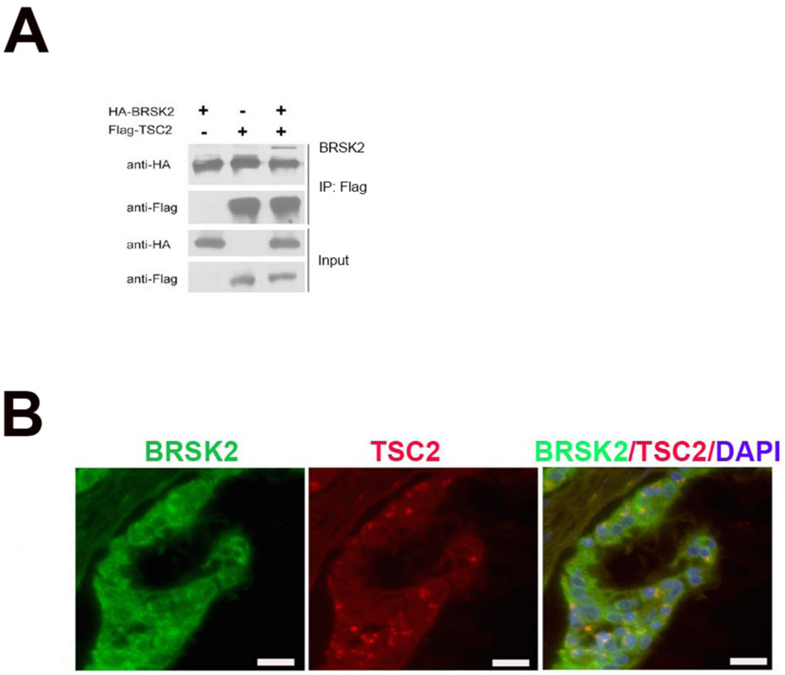

Supplementary Figure 4: BRSK2 and TSC2 interact with each other *in vitro* and colocalize in tumor tissues. (A) TSC2 and BRSK2 could interact with each other by *in vitro* immunoprecipitation assay. **(B)** BRSK2 and TSC2 were colocalized in cancer neoplastic cell of PDAC tissues. Scale bar: 10 μm.

Supplementary Table 1: Baseline clinical characteristics of PDAC and IPMN patients

		PDAC n (%)	IMPN n (%)
Gender	Male	67/102 (65.7)	16/21 (76.2)
	Female	35/102 (34.3)	5/21 (23.8)
Age	<60	40/102 (39.2)	10/21 (47.6)
	>60	62/102 (60.8)	11/21 (52.4)
Tumor location	Head	75/102 (73.5)	21/21 (100.0)
	Body-tail	21/102 (20.6)	0/21 (0)
	Entire	6/102 (5.9)	0/21(0)
Tumor stage	Tis+T1+T2	22/102 (20.8)	14/21 (66.7)
	T3+T4	80/102 (79.2)	7 (33.3)
Regional lymph status	N.A	19/102 (18.6)	4/21 (20.0)
	No	38/102 (37.3)	13/21 (65.0)
	Yes	45/102 (44.1)	3/21 (15.0)
Distant metastases	No	95/102 (93.1)	21/21 (100.0)
	Yes	7/102 (6.9)	0/21 (0)
Nerve invasion	No	27/102 (26.5)	15/19 (78.9)
	Yes	75/102 (73.5)	4/19 (21.1)
Vascular invasion	No	64/102 (62.7)	17/21 (89.5)
	Yes	38/102 (37.3)	2/21 (10.5)
Living status	lost	29/102 (28.4)	2/21 (9.5)
	live	12/102 (11.8)	10/21 (47.6)
	die	61/102 (59.8)	9/21 (42.9)

Supplementary Table 2: The correlations between BRSK2 and clinical pathological parameters in IPMN

		None	Weak	Moderate	Strong	p
		Count	Count	Count	Count	
Gender	Male	0	5	5	6	N.A
	Female	0	2	1	2	
Age	<60	0	5	2	3	N.A
	>60	0	2	4	5	
Tumor location	Head	0	7	6	8	N.A
	Body-tail	0	0	0	0	
	Entire	0	0	0	0	
Tumor stage	Tis+T1+T2	0	6	4	4	
	T3+T4	0	1	2	4	
Regional lymph status	N.A	0	1	1	2	N.A
	No	0	5	3	5	
	Yes	0	0	2	1	
Distant metastases	No	0	7	6	8	N.A
	Yes	0	0	0	0	
Nerve invasion	No	0	6	3	6	>0.05
	Yes	0	0	2	2	
Vascular invasion	No	0	6	4	7	>0.05
	Yes	0	0	1	1	
Living status	lost	0	1	0	1	HR=2.984 95% CI 0.976-14.434
	live	0	5	2	3	
	die	0	1	4	4	
